# Sex Does Not Sell: The Effect of Sexual Content on Advertisement Effectiveness and Interference with Memory for Program Information

**DOI:** 10.1177/00315125221138395

**Published:** 2022-11-06

**Authors:** Roberta-Maria Ciuvat, Adrian Furnham, Alastair McClelland

**Affiliations:** 1 Department of Experimental Psychology, 4919 University College London, London, UK; 2 6281 Norwegian Business School (BI), Oslo, Norway

**Keywords:** sexual advertising, memory, retroactive, proactive, interference

## Abstract

Does increasing the sexual content of advertisements lead, though memory processes, to greater sales? By employing a between-participants design, we aimed to explore how sexual advertising affects explicit and implicit memory, and whether it impairs memory for information preceding the commercials (retroactive interference) or following the commercials (proactive interference). We randomly assigned 182 young participants in the UK to one of two groups who watched the same TV program containing an advertisement break during which either sexual or nonsexual advertisements were shown, while brands were held constant across conditions. Participants were then tested on their explicit and implicit memory for both the advertising content and program information. Results revealed that *implicit* memory was better for nonsexual than for sexual advertisements. Unexpectedly, there was no group difference in participants’ *explicit* memory for the advertisements. Further, sexual advertising resulted in retroactive interference with program information, whereas proactive memory for program information was not impaired. We acknowledge various study limitations and discuss proposals for future research.

## Introduction

The ultimate goals of marketing communications are to increase both an awareness of goods or services and the likelihood that viewers will purchase the advertised products (Bushman, 2007). Sexual content in advertising represents the use of erotic or sexually provocative imagery, subliminal messages, or sounds to rouse consumer interest in a brand, product, or service. According to Reichert and Lambiase (2003), sexual content in advertising can be defined as verbal or visual message elements that refer to sex or activate sexual thoughts.

This study extends work by Lawrence et al. (2021) that explored whether, how, and why sex does or does not sell in advertising. Lawrence et al. (2021) also examined whether sexual appeals facilitated *implicit* memory for the brand and whether the surrounding program-type (sexual or nonsexual) impacted recall for advertisement brand name and logo to moderate the amount of recalled sexual advertisement information through congruity-effects. They found that sex enhanced memory for the advertising scene but inhibited memory for the product, essentially meaning “sex does not sell.” In this study, we examined commercials with sexual and nonsexual content to investigate whether sexual advertising (i) led to better brand and product recall, as measured by both explicit and implicit memory measures, and/or (ii) impaired memory for program information that preceded commercials (i.e., retroactive interference) or followed commercials (i.e., proactive interference). This second research topic has been of particular interest to those in advertising and marketing (Eisend & Hermann, 2019, 2020) and to sex researchers (Furnham & Mainaud, 2011; Gramazio et al., 2021; Manganello et al., 2008). Given numerous cultural changes over time, with regards to attitudes toward sex, it is important to determine whether earlier findings on this topic apply to a new consumer generation.

### Memory for Sexual Advertising and Proposed Cognitive Theories

Researchers have investigated the efficacy of sexual appeals with various outcome measures. Physiological data has suggested that individuals watching commercials of a sexual nature experience states of increased arousal and attention, as measured by galvanic skin responses (Belch et al., 1982) or increased heart rate (Sparks & Lang, 2015). In early research, a beneficial advertising effect of sexual content was demonstrated via behavioral measures of buying intentions (Reichert et al., 2001) or longer reading times when stories contained sexual versus nonsexual content (Geer et al., 1994). Using eye-tracking data, Cummins et al. (2021) found that, although sexual appeals did not increase overall attention to the ad, visual attention directed specifically to models in ads with sexual appeals was greater than visual attention directed to models in nonsexual appeals. This effect was more pronounced for men than women.

Leka et al. (2013) reported that participants rated sexual programs, some of which also contained sexual advertisements, as significantly more involving and captivating than neutral programs; this finding has implications for the surrounding context of sexual advertising. However, while several investigators concluded that sexual advertisements were better remembered than their neutral counterparts (King et al., 2015; Leka et al., 2013; Toverljani et al., 2017), others found no effect from sexual content (Bushman, 2007; Parker & Furnham, 2007) or reported contradictory results (Bushman & Bonacci, 2002). Two key meta-analyses have recently been conducted. First, in an analysis of 53 studies, Lull and Bushman (2015) showed *no beneficial effect of sexual content on advertisement recall*; in fact, as the intensity of the sexual scenes increased, viewer attitudes, memory, and purchase intentions decreased. In contrast, Writz et al. (2018) analyzed 78 experiments, and found a *positive effect of sexual content on commercial memorability* (including both recognition and recall), but they found no significant effect on either viewers’ brand recall or their attitudes towards the brand. While these two reviews illustrate a lack of clarity regarding the efficacy of sexual content in advertising, there are several important factors that may explain the contradictory nature of findings in this area.

*Evolutionary emotional arousal theory* argues that individuals have an evolutionary predisposition to attend to stimuli that are emotionally arousing for different reasons (Al-Shawaf et al., 2016). Thus, adding a sexual component to advertisements would be expected to trigger that arousal in ways that are beneficial to advertisers. However, sexual commercials require more cognitive resources from viewers than do nonsexual ones, and brands embedded in sexual commercials become peripheral to the arousing sexual content that may become the center of focus. Consequently, viewers fail to remember sexual advertisements or to recall the target brands. A further theory supporting this view is *cognitive neo-association theory* (Berkowitz, 2012), which suggests that sexual content may make viewers think about sex (i.e., remembering their own experiences), and this, in turn, reduces cognitive capacity for recalling other stimuli (i.e., the informative content of the commercial itself), disrupting the encoding of the commercial presented.

In contrast, Lang (2000) proposed a *limited capacity model,* suggesting that when individuals decide to attend to an engaging stimulus (i.e., a sexual advertisement), they allocate numerous cognitive resources to information processing that, in turn, elicit message processing. Lang’s (2000) work has recently been extended by Fisher et al. (2018) who proposed the *Limited Capacity Model of Motivated Mediated Message Processing* as an attempt to justify individuals’ motivation to attend to and remember sexual content.

The *attentional inertia theory* (Norris & Colman, 1992) proposes that the increased level of attention generated by sexual advertisements carries over to the processing of the advertisement information, making the viewer more likely to encode the information and successfully recall it at a later point. Additionally, the *Von Restorff effect* (Kishiyama & Yonelinas, 2003) predicts that, when multiple homogeneous stimuli are presented (i.e., nonsexual advertisements that individuals are used to seeing daily), the stimulus that most differs from the rest (i.e., the sexual advertisement) is more likely to be remembered.

Taken together, these varied models and theories may explain apparent contradictions between Writz et al. (2018) finding of a positive effect of sexual content on memory for commercials (both recognition and recall), but their other findings of no significant effect from sex content on either brand recall or attitudes towards the brand, which are consistent with Lull and Bushman’s (2015) meta-analysis focused on an evolutionary emotional arousal perspective. The lack of a clear consensus of the impact of sexual content on advertisement effectiveness suggests a continued need for more research that might address the actual nature of the sexual content (i.e., how stimulating, and evocative it is to the viewer) and the nature of the surrounding program.

In the current study we addressed these various issues in an to attempt to explore various past contradictory findings that included varied features of experimental designs. King et al. (2015) employed a within-subjects design, meaning that participants were exposed to both sexual and nonsexual advertisements. Bushman and Bonacci (2002) and Bushman (2005) did not adequately control other aspects of the program content, such as humor, which also has a strong impact on memory (e.g., Han et al., 2017). In the pilot stage of this study, an independent panel assessed several advertisements on the level of sexual content and on the levels of humorous and informative content, to ensure that our selected advertisements only differed in their level of sexual content. In addition, our selected TV program seemed purely informative (i.e., a documentary) rather than inclusive of sexual or humorous references. Further, we kept all brands in these advertisements constant between groups, and ensured that no participants had previously seen any of the advertisements or the TV program.

### Implicit Memory in Advertising

Implicit memory represents a behavioral change in task performance due to a prior exposure period for which there is no deliberate recollection (Schacter, 1987). In an advertising context, individuals may be shown brands they will not consciously recall or recognize later, even while their implicit memory for that brand may still be altered by the exposure. Various studies have examined this. For instance, Yang and Roskos-Ewoldsen (2007) revealed that participants’ explicit memory for advertised products improved only for products central to the program plot; but their implicit memory for the same products improved irrespective of plot relevance. Similarly, Butler and Berry (2002) showed that pairing positive valence statements with brands improved viewers’ explicit choice of those products, but this pairing had no significant effect on the viewers’ implicit choice for the products. In such implicit decision-making contexts, consumers use more peripheral cues at the expense of attribute information, they expend minimal cognitive effort and time to make the purchase choice (Dickson & Sawyer, 1990), and the intensity of their information search is drastically reduced. Therefore, it is unlikely that consumers actively engage in the conscious retrieval of brand and product information; rather, they base their choices on implicit memory and perceptual and conceptual fluency. Perceptual fluency refers to the ease of processing stimuli, based on manipulations to perceptual quality (Alter & Oppenheimer, 2009), whereas conceptual fluency refers to the ease with which the target comes to consumers' minds and pertains to the processing of meanings (Hamann, 1990).

Using implicit memory measures in advertising contexts is crucial because implicit memory is more sensitive to changes in memory-related performance than is explicit memory, and implicit memory remains stable across such conditions as delays between commercial exposure and product purchase or poor attention to the commercial (Shapiro & Krishnan, 2001). Nonetheless, prior researchers on sexual advertising have either failed to adopt implicit memory paradigms or have employed flawed methodologies. For example, by using a word-fragment task to measure implicit memory for brands, both Lee (2002) and Samu and Krishnan (2010) could only investigate perceptually implicit brand memory. While perceptual priming assesses the extent to which perceptual features of stimuli in a commercial have been processed, conceptual priming assesses how accessible information is in memory, based on conceptual retrieval cues that may better reflect the key outcomes related to consumer decision making (hence they are more ecologically valid).

Lawrence et al. (2021) investigated perceptually implicit memory in a sexual advertising context by using logos and image degradation as an implicit memory measure. Their results showed that implicit memory was weaker for sexual commercials compared to nonsexual commercials, but this finding is inconsistent with the spreading activation model (Collins & Loftus, 1975) suggesting that, at a neural level, sexual stimuli semantically prime sexual thoughts in memory and thus enhance subsequent encoding. Other studies investigating implicit memory in advertising also failed to include an assessment of awareness (e.g., Lee, 2002; Parker & Dagnall, 2009), although the measures they employed were subject to high degrees of test awareness and explicit contamination. While implicit memory is, by definition, unconscious in nature (Schacter, 1987), it should be noted that there remain several debates about whether implicit memory is conscious. Prior results interpreted as clear evidence for robust implicit priming effects might be considered questionable after considering methodological problems associated with measurement of awareness and explicit memory contamination. In the current study, we addressed these limitations by including both a conceptually implicit memory task (i.e., a timed brand generation test) and an awareness test.

### Retroactive and Proactive Memory

A further area of interest is how being exposed to sexual content may influence viewers’ memory of information seen before or after the advertisements. The nature of an advertisement may not only affect memory for the advertisement itself, but memory for the contextual program information as well. In the present study, *retroactive* memory refers to memory for program information seen before a commercial and *proactive* memory refers to memory for program information seen after a commercial.

In the context of fear-inducing content, Lang et al. (1996) showed that information acquired after seeing negative news stories was more poorly recalled than information acquired before seeing neutral news stories, indicating an emotion-induced memory impairment and retroactive interference. Similarly, Strange et al. (2003) asked participants to recall words after being exposed to negative stimuli and demonstrated retroactive memory interference.

There is also evidence that exposure to negative content can lead to proactive memory interference. Akram et al. (2018) investigated the influence of negative news stories and fearful advertisements, respectively, and they found that memory for subsequent information was worse for participants exposed to these stimuli compared those exposed to neutral ones. In contrast, Newhagen (1998) concluded that negative content can lead to proactive facilitation of memory for program information; participants’ memory for news stories was better after their exposure to negative stimuli. Therefore, the issue of whether and how emotional content influences proactive and retroactive memory for sexual commercials is still unresolved and largely unexplored.

### Current Study

In this study, we planned to investigate whether sexual content in advertisements would lead to better brand and product recall, and whether it would impair memory for program information that preceded or followed those specific advertisements. We planned to hold the commercials constant except for variations in the absence/presence of sexual content, and we sought to test conceptual implicit memory for brands to see whether embedding sexual cues in commercials would make them better primes than unembedded sexual cues. We proposed the following hypotheses:

H1: Implicit memory for target brands will be better following exposure to nonsexual (vs. sexual) advertisements.
H2: Recognition memory for advertisement information will be better following exposure to sexual (vs. nonsexual) advertisements.
H3: Memory for program information preceding sexual advertisements will be worse than memory for program information preceding nonsexual advertisements (retroactive interference).
H4: Memory for program information following sexual advertisements will be worse than memory for program information following nonsexual advertisements (proactive interference).

## Method

### Participants

An a priori analysis with G*Power 3.1 (Faul et al., 2007) revealed that a sample of 158 participants would produce adequate power (.95) to detect a medium effect size (as found in most previous studies [*f* = 0.25; Cohen, 1988]), with an assumed correlation between measures of *r* = .50. Using opportunity sampling, we recruited 182 participants (Sexual Advertisement condition: *n* = 91; Nonsexual Advertisement condition: *n* = 91) from a pool of university students via SONA. However, 27 participants reported having seen either the planned advertisement or TV program (or both) before the study and were subsequently excluded from data analysis. The remaining sample consisted of 155 participants (Sexual Advertisement condition: *n* = 81; Nonsexual Advertisement condition: *n* = 74), 133 (86%) females and 22 (14%) males, with a mean age of 18.83 years (*SD* = 1.35) and an age range of 17–29 years. Participant ethnicities were: Asian (96/155; 62%), White (50/155; 32%), Mixed/Multiple ethnic groups (2/155; 2%), and six (4%) who selected ‘Other.’

We obtained ethical approval for this research protocol from the University Ethics Committee (approval number: EP/2018/007), and all participants gave written informed consent prior to their participation in the study.

### Materials

#### Program

The TV program seen by participants was “How Dogs (Eventually) Became Our Best Friends” (Moore, 2020), a documentary broadcast by the Public Broadcasting Service (PBS) in the USA. In total, the program was 9 minutes and 14 seconds long; it was divided into two parts (the former lasting for five minutes and the latter lasting for 4 minutes and 14 seconds) between which we presented the advertisements break.

#### Advertisements

In a pilot study, we presented 24 advertisements (four advertisements for each of six different brands) to an independent panel of 10 consenting students (5 females, five males, aged 18–25 years) who rated them on three 5-point Likert scales (1 = not at all; 5 = very) to indicate how sexy, amusing, and informative they perceived the ads to be. This group was chosen because they were like the participants we planned to use in the study. After eliminating any outliers (i.e., advertisements that drew dramatically different responses from this pilot participant sample) we selected advertisements that were judged to be the most and least sexual and kept the brands constant across conditions. The brands and product categories included in the advertisement break were Carl’s Jr. (burgers), Calvin Klein (jeans), Jean Paul Gaultier (fragrance), and Nivea (toiletries). For each brand, we selected an advertisement with sexual content and an advertisement with nonsexual content. Table 1 describes the content of each advertisement.

**Table 1.** Content of Advertisements.

Brand	Sexual advertisement	Nonsexual advertisement
Carl’s Jr	Women wearing scanty clothing on the beach and touching each other’s hips; men looking at them while eating Carl’s Jr. Cheeseburgers	Men eating Carl’s Jr. Cheeseburgers
Calvin Klein	Couples wearing Calvin Klein jeans while kissing, cuddling, and touching each other’s bodies	Man and woman wearing different types of Calvin Klein jeans
Jean Paul Gaultier	Men and women wearing scanty clothing; couple kissing	Ingredients and scents used in a Jean Paul Gaultier perfume
Nivea	Woman showering using a Nivea product and touching her naked body; man caressing the woman’s shoulders	Demonstrating how a Nivea product can protect one’s skin from ultraviolet radiation

The commercials had never been broadcast in the UK, and they were reasonably matched in length, resulting in final video compilations of 1′55″ duration (nonsexual condition and 2′25″ (sexual condition), respectively. This duration difference was not expected to impact results. The order of the commercials within the final video compilations was fixed, and the brand order was the same in both conditions. While this could have led to a memory recency effect, there was no evidence of this occurring.

### Brand Generation Task

This task served as a conceptual implicit memory test. A product category appeared on a computer screen, and participants were asked to provide the very first brand name fitting that category that came to their mind (e.g., for “car” they could indicate BMW) by typing that name into a box. Participants were awarded one point for each target brand indicated, and, thus, their scores ranged from 0 to 4. There was a time limit of 10 seconds for each product category, after which the screen advanced. There were 20 product categories included in this task (four targets and 16 foils). The time limit was imposed to ensure participants provided the very first brand name they thought of; unlimited time may have undermined the validity of this implicit memory measure.

### Questionnaires

#### Awareness Questionnaire

Participants completed an awareness questionnaire taken from Northup and Mulligan (2013) that included five increasingly specific questions to determine their awareness of the aim of our conceptual implicit memory task while performing it, and whether they had engaged in intentional retrieval. Those participants who indicated awareness of the connection between the brand names they had read earlier and the brand generation task while performing it (question four) were classified as “test aware.” These participants were further classified as engaging in either intentional or unintentional retrieval, dependent upon their answer to question five. Importantly, this categorization of participants has shown good validity and reliability in prior research (Barnhardt & Geraci, 2008).

We took two steps to minimize the likelihood of participants becoming aware of the aim of the implicit memory test. First, we included 16 foil product categories in the brand generation task. Second, before beginning the study, participants were told that they would be completing two different, independent tasks (i.e., the timed brand generation test followed by the other questionnaires). Northup and Mulligan (2013) suggested that presenting the brand generation test as a separate research task significantly reduced awareness and explicit memory contamination levels.

#### Recognition Memory Questionnaire

Participants completed four multiple-choice tests for logo recognition, each including one of the target logos and five foils. The four tests appeared in random order, but the order of the logos within each test was fixed. The target logo and the foils were matched for their product category (i.e., all logos in one multiple-choice test were from brands selling burgers, jeans, fragrance, or toiletries, respectively) and for their chromatic features (i.e., all logos in one multiple-choice test had the same colours). Participants could obtain a score between 0 and 4.

Next, participants completed one multiple-choice test for product categories recognition, including 20 product categories (i.e., four target categories plus 16 foils). The 20 product categories appeared on the test list in a fixed order. Participants could again obtain a score between 0 and 4. Their final recognition memory score was computed by summing their logo recognition and product category recognition scores, and thus ranged from 0 to 8.

#### Retroactive and Proactive Memory Questionnaires

These questionnaires consisted of five questions each on TV program information that either preceded (retroactive) or followed (practive) the advertisement break. The questions were based on a list of the most notable features seen in the TV program, matched for difficulty by showing high agreement (95%) about their difficulty between an independent judge and the first author. Retroactive and proactive memory scores could range from 0 to 5.

### Procedure

This study was designed and conducted virtually using the experiment builder, Gorilla (Anwyl-Irvine et al., 2020), and it was live between November 26, 2020 and January 5, 2021. As noted, participants were told that the study included two different, independent tasks that must both be completed to gain the 0.5 point course credit. They then gave their consent and were randomly assigned by the experiment builder to one of the two experimental conditions (sexual or nonsexual), on a 1:1 ratio. This randomization was successful, in that there were no significant group differences in age or ethnicity. Participants watched the first half of the TV program, followed by the advertisements break in which they saw four commercials. All commercials had either sexual or nonsexual content, conditional upon the experimental group to which they were shown. After the advertisement break, participants watched the second half of the TV program. Once the TV program had finished, participants were given the brand generation task. After completing the task, participants were asked to complete the awareness questionnaire that categorized them as test aware versus test unaware, and as engaging in intentional versus unintentional retrieval. Finally, participants completed the recognition, retroactive, and proactive memory questionnaires in that order and with no time limit. They were later debriefed about the aims of the study.

## Results

Four scores were computed for each participant: a score for how many target brands they generated during the implicit memory task, a recognition memory score for advertisement information they recognized, and both a retroactive, and proactive memory score for program information. There was no missing data and statistical significance was set at *p* < .05. All scores were converted to percentages prior to analysis to make for easier comparisons.

### Awareness

Based on responses to the awareness questionnaire (Northup & Mulligan, 2013), participants were categorized as test aware or test unaware during the conceptual implicit memory test. Of the 155 responses analyzed, 52 participants (33.5%) were unaware and 103 (66.5%) were aware of the relationship between the study and the aim of the implicit memory task. Of those who were aware, 74 (72%) reported not engaging in intentional retrieval and 29 (28%) reported engaging in intentional retrieval.

### Correlations Between Memory Measures

Pearson product-moment correlations between the four memory measures are presented in Table 2. There was a moderate positive correlation between the two measures for memory of the advertisements (measures 1 and 2), *r*(153) = .432, *p* < 0.01, and between the two measures for memory of the TV program (measures 3 and 4), *r*(153) = .518, *p* < 0.01.

**Table 2.** Correlations Between Memory Measures (*N* = 155).

Memory measure	1	2	3	4
1. Implicit memory	–			
2. Recognition memory	.432**	–		
3. Retroactive memory	.161*	.333**	–	
4. Proactive memory	.334**	.435**	.518**	–

*p < 0.05. **p < 0.01.

### Memory for Advertisement Information

The means (and standard deviations) for the measures assessing memory for advertisement information (i.e., percentage of target brands generated and recognition memory) under the two different advertisement content conditions (i.e., sexual and nonsexual) are presented in Table 3.

**Table 3.** Percentage of Target Brands Generated (Implicit Memory) and Advertisement Information Recognized as a Function of Advertisement Content (*N* = 155).

	Advertisement content		
	Sexual (*n* = 81)	Nonsexual (*n* = 74)	
Memory measure	*M* (*SD*)	*M* (*SD*)	d
Implicit memory	21.96 (20.20)	32.04 (20.66)	0.49
Recognition memory	79.00 (18.72)	77.38 (21.38)	0.08

To assess the effect of sexual content on memory for the commercials, we conducted a one-way multivariate analysis of variance (MANOVA), with advertisement content (sexual vs. nonsexual) as the independent variable and the two advertisement memory measures as dependent variables. There was a significant effect of advertisement content on memory for advertisements, as indicated by Pillai’s trace statistic: V = 0.08, *F*(2, 152) = 6.21, *p* = 0.003, η_p_^2^ =.076. A Pearson product-moment correlation coefficient computed to assess the relationship between the two advertisement memory measures revealed a moderate positive correlation (see Table 2). Koslowsky and Caspy (1991) recommended that further step-down analyses of covariance (ANCOVA) should be conducted when the dependent variables included in MANOVAs are significantly correlated.

For all ANCOVAs, advertisement content (sexual vs. nonsexual) was the independent variable. In the first ANCOVA, the proportion of target brands generated during the implicit memory task was the dependent variable, and recognition memory for advertisement information was the covariate. The covariate was significant, *F*(1, 152) = 33.97, *p* < 0.001, η_p_^2^ = .183, and there was also a significant effect of advertisement content on implicit memory test performance, *F*(1, 152) = 11.38, *p* = 0.001, η_p_^2^ = .070. Critically, participants assigned to the nonsexual advertisements (*M*_*adj*_ = 32.04) generated significantly more target brands during the brand generation task than participants assigned to the sexual advertisements (*M*_*adj*_ = 21.96). Thus, our first hypothesis was supported.

In a follow-up analysis, we investigated whether the pattern of results differed between aware and unaware participant sub-samples. For the unaware participants there was no significant difference in the proportion of target brands generated between those exposed to sexual (*M* = 16.91) versus nonsexual (*M* = 15.28) advertising content, *F* < 1. However, among aware participants, the target brand generation difference was significant between participants exposed to nonsexual commercials (*M* = 38.39) and those exposed to sexual commercials (*M* = 24.47), *F*(1, 101) = 14.07, *p* < 0.001, η_p_^2^ = .122. A two-way ANOVA conducted on the aware sub-sample with the implicit task performance as the dependent variable and the experimental condition and presence/absence of intentional retrieval as independent variables revealed no significant interaction between the experimental condition and whether participants engaged in intentional retrieval, *F* < 1. Additionally, engaging in intentional retrieval had no effect on the proportion of target brands generated, *F*(1, 99) = 2.60, *p* = 0.11, η_p_^2^ = .026.

In the second ANCOVA, recognition memory for advertisement information was the dependent variable, and the proportion of target brands generated during the implicit memory task was the covariate. The covariate was significant, *F*(1, 152) = 33.97, *p* < 0.001, η_p_^2^ = .183, but there was no significant effect of advertisement content on recognition memory for advertisement information, *F* < 1. Participants assigned to the sexual condition (*M*_*adj*_ = 79.00) did not recall advertisement information significantly better than those assigned to the nonsexual condition (*M*_*adj*_ = 77.38). Therefore, our second hypothesis was not supported.

### Memory for Program Information

The participants’ means (and standard deviations) on measures assessing memory for program information (i.e., retroactive and proactive memory) under the two advertisement content conditions are presented in Table 4.

**Table 4.** Percentage of TV Program Information Recognized as a Function of Advertisement Content (*N* = 155).

	Advertisement content		
	Sexual (*n* = 81)	Nonsexual (*n* = 74)	
Memory measure	*M* (*SD*)	*M* (*SD*)	d
Retroactive memory	47.45 (27.25)	55.63 (28.28)	.34
Proactive memory	43.99 (26.06)	45.36 (31.94)	.13

To investigate the effect of sexual content on memory for program information, we conducted a MANOVA with advertisement content (sexual vs. nonsexual) as the independent variable and the two program information memory measures as the dependent variables. There was a significant effect of advertisement content on memory for advertisements, as indicated by Pillai’s trace statistic: V = 0.05, *F*(2, 152) = 3.59, *p* = 0.03, η_p_^2^ = .450. However, the relationship between the two program memory measures was again significant (see Table 2); so, following Koslowsky and Caspy’s (1991) recommendation, we conducted two separate ANCOVAs.

In the first ANCOVA, retroactive memory was the dependent variable and proactive memory was the covariate. The covariate was significant, *F*(1, 152) = 52.29, *p* < 0.001, η_p_^2^ = .256, and there was also a significant effect of advertisement content on retroactive memory for TV program information, *F*(1, 152) = 4.41, *p* = 0.037, η_p_^2^ = .028. Critically, participants exposed to the nonsexual commercials (*M*_*adj*_ = 55.63) recalled the first half of the TV program significantly better than those exposed to the sexual commercials (*M*_*adj*_ = 47.45). Therefore, the sexual content of advertisements had a detrimental effect on participants’ retroactive memory for the TV program, and our third hypothesis was supported. However, it should be noted that the effect size was small, particularly when compared to the other hypotheses.

In the second ANCOVA, proactive memory was the dependent variable and retroactive memory was the covariate. The covariate was significant, *F*(1, 152) = 52.29, *p* < 0.001, η_p_^2^ = .256, but there was no significant effect of advertisement content on proactive memory for TV program information, *F* < 1. There was no significant difference in participants’ ability to recall the second half of the TV program information following exposure to sexual (*M*_*adj*_ = 43.99) or nonsexual (*M*_*adj*_ = 45.36) commercials. Thus, our fourth hypothesis was not supported.

## Discussion

Our first hypothesis that during the implicit memory task, participants exposed to nonsexual advertisements would generate target brands more frequently than those exposed to sexual advertisements, was supported. Our second hypothesis was not supported. Brand priming was no more successful when participants did not encounter engaging and attention-grabbing sexual stimuli in the commercials; thus including sexual content in advertisements did not impair implicit memory for brands and had no effect on logo and product category recognition. These findings are inconsistent with Lang’s (2000) *limited capacity model* in which individuals who attend to engaging stimuli allocate numerous cognitive resources to information processing that elicit message processing and results in greater memory storage capacity and stronger memory traces. They are also inconsistent with *attentional inertia theory* (Norris & Colman, 1992) in which the increased level of attention generated by sexual advertisements are expected to carry over to the processing of advertisement information, making the viewer more likely to encode the information and successfully recall it later.

The findings are more consistent with the *evolutionary emotional arousal* perspective in which Lull and Bushman (2015) argued that our cognitive resources are highly involved in processing the sexual content embedded in sexual advertising. However, because they are limited, other information, such as the logo or the product category receive less processing and are less well remembered. The failure to find recognition memory superiority for sexual advertisements supports Lull and Bushman’s (2015) meta-analysis and those previous studies that found no effect of sexual content on commercial memorability (e.g., Bushman, 2007; Parker & Furnham, 2007).

Our finding of implicit memory superiority for nonsexual advertisements is in line with Lawrence et al. (2021) in which logos and image degradation were used as an implicit memory measure. Moreover, Shapiro and Krishnan (2001) and Law and Braun-LaTour (2004) suggested that implicit memory measures are more ecologically valid and reflective of real-world consumer behavior than are explicit memory measures. Thus, our findings add to the literature by demonstrating that sexual appeals in advertising have a detrimental effect not only on implicit brand recognition (Lawrence et al., 2021), but also on implicit brand recall.

A potential reason for not finding superior explicit memory for sexual advertisements could be that the sexual advertisements we selected were not “sexual” enough. For example, King et al. (2015) found a significant effect of sexual advertising for skin products when all actors were completely naked. Thus, a Von Restorff effect may have influenced memory accordingly. Additionally, a review of adolescent sexuality and the media (Gruber & Grube, 2000) warned about young people’s exposure to sexual content through television and other electronic media, noting that these exposure rates might significantly increase over the following decade. In fact, a recent meta-analysis (Madigan et al., 2018) that included studies from 1990 to 2016 investigated the prevalence of unwanted online exposure and solicitation of sexual media among youth and revealed that 20% of participants were exposed to unwanted sexual images online and 11% had received requests to engage in sexual activities. Hence, the increasing ease of access to sexual material over time might mean that the young adults are no longer surprised or distracted by sexual content in broadcast media. These factors may have led participants in this study not to pay significantly more attention to the sexual commercials, resulting in similar recognition rates for sexual and nonsexual content.

### Memory for Program Information

Our third hypothesis was supported, but our fourth hypothesis was not. The use of sexual content in commercials caused the memory of some previously observed information to be impaired, whereas this same sexual content in commercials had no effect on participants’ memory for information following the commercials. Possibly, retroactive and proactive memory interference are age dependent (Darby & Sloutsky, 2015; Kail, 2002; Lewis-Peacock & Norman, 2014), and our relatively young sample (*M*_*age*_ = 18.83 years) showed retroactive interference, while already having developed adequate proactive memory-related cognitive resources to withstand proactive interference. This alternative is further supported by a neural perspective suggesting that distinct neural mechanisms may modulate proactive and retrospective memory interference effects. While proactive interference is thought to be linked to executive functions such as active or working memory (Morton & Munakata, 2002) and resistance to proactive interference has been associated with prefrontal cortical activity (Jonides & Nee, 2006), the hippocampus may play a more important role in resistance to retroactive interference (Kuhl et al., 2010). Experimental advertising research that combines neural (e.g., neuroimaging techniques) and cognitive/behavioral data would further advance our understanding.

An alternative account relates to the length of the TV program selected for the current study. In previous experiments demonstrating both retroactive and proactive interference effects from negative stimuli, the news stories were, on average, three minutes long. This length is three times shorter than the length of the TV program used in the current study. Hence, proactive interference from sexual content may be time-sensitive, with effects only evident for information that more immediately follows participants’ exposure to the sexual content. Overall, our findings are consistent with extant research on fear in advertising which has demonstrated that being exposed to negative news stories (Lang et al., 1996) or emotional words (Strange et al., 2003) results in emotion-induced memory impairment and retroactive interference. However, they are inconsistent with previous findings of proactive interference (Akram et al., 2018) or facilitation (Newhagen & Reeves, 1992) caused by fearful stimuli. They also do not support the transfer hypothesis (Moorman et al., 2012), which states that sexual imagery (i.e., the sexual advertisements) is more likely to arouse attention and increase memory for the incoming stimuli (i.e., proactive facilitation for the sexual group). The current study adds to the literature by highlighting that individuals exposed to sexual advertisements are apt to have poorer memory for information preceding the advertisements but no impairment in memory for information following the advertisements.

### Limitations and Directions for Further Research

Among this study’s limitations was the prevalence of women versus men in our participant sample, perhaps limiting a generalization of these findings to the general population. Additionally, as retroactive and proactive memory interference have been found to be age-dependent, it will be important to study these phenomena with participants of different ages. Because we used “real” as opposed to “experimental” data neither our ads nor our TV program were perfectly matched. Additionally, we did not check for the extent to which our participants thought that the advertisements contained sexual content. Future investigators might further examine the effects of greater and lesser intensity of sexual imagery and content on memory, though this research is made more difficult in the context of governmental oversight and needs for human participant protection.

## Conclusion

In sum, our results in this study suggest that *sex does not sell,* a finding that is consistent with that of earlier studies (Bushman & Bonacci, 2002) and meta-analyses (Lull & Bushman, 2015). Sexual advertising did not lead to enhanced recognition memory for the commercials, and implicit memory was better for the nonsexual advertisements, confirming the findings from Lawrence et al., (2021) who similarly found that sexual advertising led to increased memory for the advertisement scene itself at the expense of recalling the brand.

While sexual advertising resulted in retroactive interference of memory for TV program information, proactive memory for TV program information was not impaired by it. Overall, these results lend support to the proposal that modern marketers should not rely on the inclusion of sexual content as an effective communication persuasion strategy; rather, they should target consumers’ implicit memory with neutral messages. TV program producers should also exercise caution about showing commercials with sexual content during ad breaks, as this has implications for retrospective memory of program information.
